# Evidence-Based Paediatric Oncology: Second Edition

**DOI:** 10.1038/sj.bjc.6604147

**Published:** 2008-01-08

**Authors:** J Anderson

**Affiliations:** 1 Institute of Child Health and Great Ormond Street Hospital, London, UK

Pinkerton, Shankar and Matthay have edited a second edition of a text, which is designed as a clinical management resource for clinicians involved in the care of children with cancer. Roughly, equal space is afforded to haematological and nonhaematological malignancy, which is a fair reflection of the relative incidences of those diseases.

The format of the book remains as an investigation of the evidence supporting currently perceived best practice. Each chapter follows the same format. To take Wilms' tumour as an example, written by Daniel Green, there is a short overview of the basics of the treatment of the disease, followed by 13 separate critiques of significant clinical studies, the results of which form the foundation of the current best practice. For each study, there is sufficient information of methodology to allow the readers to interpret for themselves the likely significance of the work. There is also a short boxed conclusion for each clinical trial, which allows the reader to quickly identify some published work that would lead to the appropriate literature. This could be used (and has been in the case of this reviewer) as a textbook resource for starting a literature search when faced by a real clinical dilemma and wanting to review the published literature again.

There is 1 chapter per clinical issue, 10 for solid tumours, 10 for leukaemia and 2 for issues in supportive care. For the solid tumours, this equates to a chapter for each common histology. The leukaemia chapters are written by Judith Chessells and Vaskar Saha and cover separate issues. For acute myeloid leukaemia, there are separate chapters for comparison of induction regimens, role of autografts and role of maintenance chemotherapy. For acute lymphoblastic leukaemia, the three chapters review induction regimes, CNS prophylaxis and maintenance therapy. The supportive care chapters review the use of haemopoietic colony-stimulating factors and of cardioprotection.

What this book does not do is attempt to provide the reader with a detailed understanding of the diseases encountered in paediatric oncology. It is assumed that there is a good working knowledge of the main histologies in terms of presentation, genetics and overall management.

Throughout, the book is illustrated with figures of trial design usually married to the survival curves for the different treatment groups. It is likely to continue to be a valuable resource to clinicians in the field for years to come. The pace of change in oncology is relatively slow, and results from trials in the 80s and 90s in many cases continue to form the basis of many of the ‘givens’ in the filed. The book is particularly helpful in providing an overview of independent and independently published trials, which might not otherwise be juxtaposed in the reader's mind.

